# Right Care, First Time: Developing a Theory-Based Automated Protocol to Help Clinically Stage Young People Based on Severity and Persistence of Mental Illness

**DOI:** 10.3389/fpubh.2021.621862

**Published:** 2021-08-27

**Authors:** Frank Iorfino, Vanessa Wan Sze Cheng, Shane P. Cross, Hannah F. Yee, Tracey A. Davenport, Elizabeth M. Scott, Ian B. Hickie

**Affiliations:** Brain and Mind Centre, The University of Sydney, Sydney, NSW, Australia

**Keywords:** mental health, eHealth, health informatics, clinical decision support, health information technologies

## Abstract

Most mental disorders emerge before the age of 25 years and, if left untreated, have the potential to lead to considerable lifetime burden of disease. Many services struggle to manage high demand and have difficulty matching individuals to timely interventions due to the heterogeneity of disorders. The technological implementation of clinical staging for youth mental health may assist the early detection and treatment of mental disorders. We describe the development of a theory-based automated protocol to facilitate the initial clinical staging process, its intended use, and strategies for protocol validation and refinement. The automated clinical staging protocol leverages the clinical validation and evidence base of the staging model to improve its standardization, scalability, and utility by deploying it using Health Information Technologies (HIT). Its use has the potential to enhance clinical decision-making and transform existing care pathways, but further validation and evaluation of the tool in real-world settings is needed.

## Introduction

Most mental disorders emerge before the age of 25 years and result in considerable burden of disease (1, 2). The early onset of disorders often has lifelong impacts even if the disorder is subthreshold or has remitted, so effective mental health care during this period is critical to reduce their burden (3, 4). While youth mental health services have improved access to care (5–8), many services struggle to manage high demand and have difficulty matching individuals to timely interventions due to the heterogeneity of disorders. Together these challenges perpetuate a vicious cycle between health service inefficiencies and poor treatment outcomes at high costs to the health system and society (9–12).

The increased adoption and development of HITs in mental health emphasize their growing importance in the mental health services landscape (13–15). Using HITs for assessment, triage and referral is an area of particular interest due to the scalability and standardization of technologies (16). Though, the utility of triage protocols depends on having a heuristic for allocating care appropriately, which accounts for the complexities of emerging mood and psychotic disorders among youth (17). For this the clinical staging model for youth mental health may be particularly useful as a validated and transdiagnostic framework that aims to deal with the heterogeneity of disorders based on the persistence and severity of their symptoms and syndromes (18). The application of the clinical staging model using HITs could improve its scalability and serve as a useful guiding tool for clinicians making treatment decisions and service managers deciding how to allocate resources to provide the most effective care pathways. The objective of this paper is to describe the translation of the clinical staging model into a decision support tool using HITs.

### Clinical Staging for Youth Mental Health

Young people experiencing mental illness vary along a continuum by factors including severity, duration of symptoms, and illness course (e.g., first episode vs. recurrent illness). Clinical staging is a framework which deals with clinical heterogeneity by using these factors to distinguish between young people in the early subthreshold phases of illness from those who have reached full threshold for major, discrete, and persistent or recurrent disorders. The clinical staging model is summarized in Table 1, and a detailed description of its development, validity and utility can be found in previous publications (18–22).

**Table 1:** Clinical stages for youth mental health (18).

**Clinical stage**	**Definition**
Stage 1a	Non-specific symptoms, mild to moderate symptom severity and only recent or mild impacts on social, educational, or occupational functioning
Stage 1b	Attenuated syndromes, with more specific anxiety, depression, mania, or psychosis symptoms of a moderate to severe severity and moderate to severe impacts on social, educational, or occupational functioning
Stage 2	Relatively more “discrete” disorders, with clear depressive, manic, psychotic, or mixed syndromes that persist over time, and clear, major impacts on social, educational, or occupational functioning
Stage 3	Discrete disorders that have persisted for at least 12 months following reasonable course of treatment or recurred after a complete recovery period of at least 3 months, with associated deterioration in social, educational, or occupational functioning
Stage 4	Chronic, severe, and unremitting illness that has persisted without remission for at least 2 years, with associated marked deterioration in social, educational, or occupational functioning

**Figure 1:**
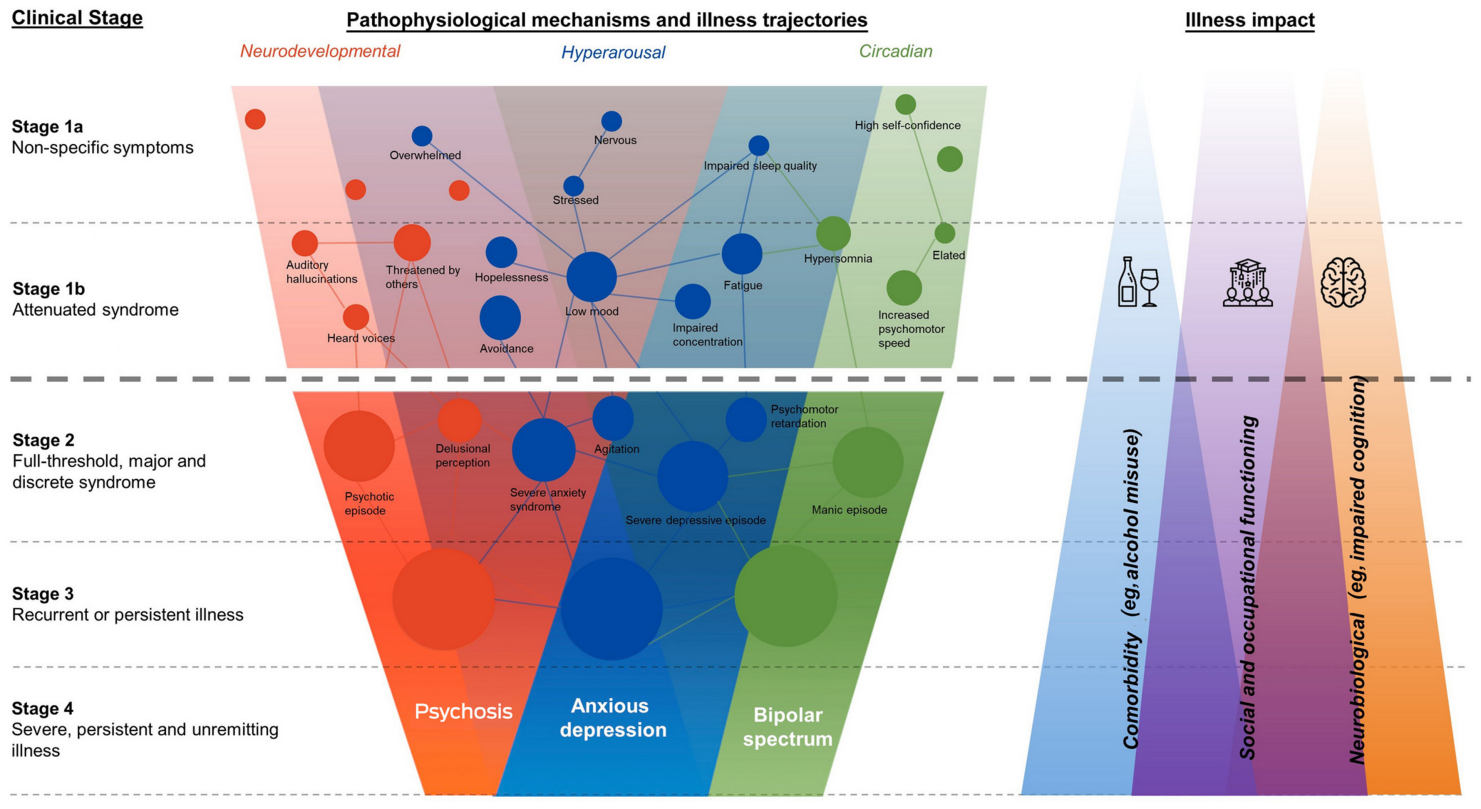
Transdiagnostic framework of clinical stages overlapping pathophysiological domains, and theorized magnitude of illness impacts (19).

The clinical staging model is supported by a range of clinical and neurobiological validation studies. The construct validity of the model is supported by the high rates of agreement for classifying clinical stage across independent expert raters (18), and longitudinal work supports the differential rates of progression from earlier to later stages of anxiety, mood, psychotic, or comorbid disorders (23)—a key assumption of the model. Clinical stage has also been associated with neurobiological change [e.g., white brain matter; (24), differences in neuropsychological performance (25), and sleep disruption (26)]. These objective features characterize major demarcation points in adolescent-onset mood and psychotic syndromes, which are consistent with the clinical staging models assumptions about illness progression and severity (Figure 1).

The clinical staging model lends itself to allocating different levels of care and providing early intervention to slow or prevent the emergence and recurrence of these syndromes (27). Young people at later clinical stages will typically (though not always) require more intensive mental health care (19). While stages 1a and 1b represent subthreshold syndromes, and if left untreated, they have the potential to progress to more debilitating and persistent mental illness. Stage 1b in particular describes a relatively more severe state of attenuated syndrome that is associated with a higher risk of progressing to the development of more persistent and crystallized symptom clusters characteristic of discrete disorders (23). Young people at stage 1b are therefore recommended for more frequent clinical follow-up compared to those at stage 1a, as well as lengthier monitoring. This translates to a more intensive allocation of service resources (17). Therefore, identifying whether young people presenting to mental health services are at stage 1a, stage 1b, or beyond (Stage 2+) is of paramount importance for effective early intervention and secondary prevention.

Importantly, and as is the case with other clinical staging models (e.g., cancer), once placed on the mental illness continuum and assigned a clinical stage, young people cannot move back to an earlier stage, even in case of remission. As guidelines and standards of care are developed around the various clinical stages, this may provide a more accessible frame of reference for mental health consumers to quickly develop a shared understanding of their mental health with their clinician as well as an idea of what to expect in treatment. Ultimately, this framework has the potential to assist mental health services in resource (particularly human resource) management and in ensuring that all young people presenting to mental health services receive the most appropriate care for their personal circumstances that minimizes their risk of illness progression: “right care, first time.”

## Developing an Automated Clinical Staging Protocol for Hits

This paper extends previous work on developing a clinical decision-making protocol based on the clinical staging framework (18, 23), by presenting the basic structure of an automated version of this protocol (Algorithms 1 and 2). This automated protocol was developed as a feature of a HIT that aimed to support mental health services by (among other things) automating intake processes, including conducting a multidimensional initial assessment by collecting demographic data, administering psychometric scales, and providing real-time feedback about results. This HIT is currently used in multiple youth mental health services across Australia (28).

The multidimensional initial assessment is the first step of a proposed model for youth mental health service delivery that aims to deliver highly personalized and measurement-based care (19, 29). In this step, a comprehensive assessment spanning a wide variety of biopsychosocial health domains is conducted in order to gather enough information to allocate the appropriate intensity and type (e.g., online CBT, clinician-delivered CBT, “non-mental health” interventions focusing on returning to school) of care (27, 29). By using clinical staging principles to distill a young person's scores on key domains into a suggested clinical stage, this automated protocol aims to provide another source of information for clinicians to consider when making key treatment decisions (including shared decision-making with the young person).

We present this automated protocol in algorithm format (in pseudocode) for ease of understanding and to illustrate how the clinical staging model has been translated. The translation of this model was led by FI, SC, and IH. The process involved collating all previously published works on clinical staging criteria and supporting evidence and identifying the critical differentiating features from these publications. These differentiating features were best matched to the self-report items available in the HIT. This process involved wider consultation with youth mental health clinicians practicing in the application of clinical staging for young people, until the algorithm could be refined to maximize face validity.

We focused on distinguishing between young people at stages 1a, 1b, and 2+ at this initial phase of development as these clinical stages are the most relevant for early intervention and prevention. The protocol aims to automate two critical decision points associated with clinical staging (19, 23) and is presented in Table 2. The first is to determine whether there is any clear evidence of at least one full-threshold, major, discrete, and persistent or recurrent syndrome. This decision aims to distinguish between stage 2+ disorders and stage 1 (1a or 1b) disorders. The second is to then determine, of those among the stage 1 group, whether the syndrome is non-specific or attenuated. This decision aims to separate stage 1a and stage 1b syndromes.

**Table 2:** Pseudocode for the translation of clinical staging decisions into an algorithm.

**Algorithm 1: clinical staging algorithm** //Apply formula to determine if young person meets conditions of being rated stage 2+ **IF** *Social and occupational function rating indicates ongoing and major impact on functioning* **AND** ( * Clear manic syndrome (not just symptoms)* **OR** *Clear psychotic syndrome (not just symptoms)* **OR** *Clear severe depressive syndrome* **OR** *Clear severe anxiety syndrome* **OR** *Previous Hospitalization for mental ill-health* **OR** *Significant and ongoing comorbid syndromes (e.g., substance misuse, eating disorders, personality)* ) **THEN** assign ‘Stage 2+' //Apply formula to determine if young person meets conditions of being rated stage 1b syndrome **ELSE IF** *Social and occupational function rating indicates moderate to severe impact on functioning* **AND** ( * Specific and more severe anxiety syndrome (e.g., avoidance)* **OR** * Moderate depression syndrome without features indicative of stage 2+* **OR** * Hypomanic or attenuated psychotic symptoms as part of mood or anxiety syndrome* **OR** * Significant comorbid syndromes (e.g., substance, eating disorders, personality disorders)* ) **THEN** assign ‘Stage 1b' //Young person does not meet criteria for stage 2+ or stage 1b, therefore assign stage 1a **ELSE** assign ‘Stage 1a'

Each of the evaluation criterion (text in italics) found in the algorithm 1 are underpinned by a secondary algorithm which evaluates the raw data to ascertain a result. Table 3 presents an example of a secondary algorithm used to evaluate a young person's depression results to determine the appropriate flag for algorithm 1. The examples in this table were chosen to show the versatility with which these secondary algorithms can feed into algorithm 1, and how the clinical staging model has been operationalized using scale thresholds, individual items and symptom severity in other mental health domains (e.g., mania-like experiences and psychotic-like experiences).

**Table 3:** Detailed example for the translation of self-report data into clinical staging decisions.

**Algorithm 2: specific clinical staging algorithm for depressive syndromes** //Apply formula to determine if young person meets conditions of a stage 2+ depressive syndrome **IF** //Evaluate cut-offs for ‘Severe' depressive symptoms (QIDS ≥ 21 **OR** PHQ-9 ≥ 20) **AND** //Evaluate features indicative of more severe syndromes **A: Two or more symptoms indicative of a severe syndrome** ( * QIDS – either of the slow/restless items ≥ 3* **OR** *//psychomotor retardation/agitation* * PHQ-9 – slow/restless item ≥ 3* **OR** *//psychomotor retardation/agitation* * QIDS – highest score on sleep items = 3* **OR** *//severe circadian dysfunction* * PHQ-9 – sleep item = 3* **OR** *//severe circadian dysfunction* * QIDS – concentration/decision making item ≥ 3* **OR** *//major cognitive impairment* * PHQ-9 – trouble concentrating item ≥ 3* **OR** *//major cognitive impairment* * QIDS – Energy level item ≥3* **OR** *//severe energy disruption* * PHQ-9 – tired or little energy item ≥3 //severe energy disruption* ) **AND** **B: At least one other specific feature indicative of a severe syndrome** ( * Probable hypomanic episodes* **OR** * Probable psychotic symptoms* **OR** * Severe suicidality* **OR** * Probable comorbidity (e.g., anxiety disorder, personality disorder, eating disorder)* **OR** * Probable substance misuse* **OR** * Flag for early onset, previous severe episode, treatment resistance or recurring illness* ) **THEN** assign ‘Stage 2+ - Clear severe depressive syndrome' //Apply formula to determine if young person meets conditions of a stage 1b depressive syndrome **ELSE IF** //Evaluate cut-offs for ‘Moderate' depressive syndrome (QIDS ≥ 11 **OR** PHQ-9 ≥ 10) **AND** //Evaluate features indicative of more moderate syndromes **A: Two or more symptoms indicative of a moderate syndrome** ( * QIDS – either of the slow/restless items ≥ 2* **OR** * PHQ-9 – slow/restless item ≥ 2* **OR** * QIDS – highest score on sleep items = 2* **OR** * PHQ-9 – sleep item = 2* **OR** * QIDS – concentration/decision making item ≥ 2* **OR** * PHQ-9 – trouble concentrating item ≥ 2* **OR** * QIDS – Energy level item ≥2* **OR** * PHQ-9 – tired or little energy item ≥2* ) **AND** **B: At least one other specific feature indicative of a moderate syndrome** ( * Possible hypomanic episodes* **OR** * Possible psychotic symptoms* **OR** * Severe suicidality* **OR** * Possible comorbidity (e.g., anxiety disorder, personality disorder, eating disorder)* **OR** * ossible substance misuse* ) **THEN** assign ‘Stage 1b—Moderate depression syndrome; **ELSE** assign ‘Stage 1a—Non-specific, mild depressive syndrome'

Multiple scales measuring the same construct, such as the Quick Inventory of Depressive Symptomatology [QIDS; (30)], or the Patient Health Questionnaire-9 [PHQ-9; (31)], which measure depressive symptoms can be added as conditions. This increases the versatility of the protocol (and the HIT) since it can be simultaneously used by multiple services employing different psychometric scales. Should they fit clinical staging criteria, responses to individual scale items can also be added to the secondary algorithms. For example, the QIDS items “*Feeling slowed down*” and “*Feeling restless*” (30) are conceptually similar to the PHQ-9 item “*Moving or speaking so slowly that other people could have noticed. Or, the opposite—being so fidgety or restless that you have been moving around a lot more than usual*” (31). As endorsements of these items can indicate specific functional or circadian disruptions differentially associated with clinical staging (compared to other QIDS and PHQ-9 items or total scores), adding these as flag conditions can allow more targeted identification of young people at higher risk of worsening mental health outcomes. Specifying the exact degree of endorsement (e.g., a response of “2” or “3” on a 4-point Likert scale ranging from “0” to “3” where “0” indicates no impairment) could further increase precision.

While the secondary algorithm allows for the incorporation of multiple scales measuring the same construct, they cannot be assumed to be psychometrically equivalent to each other. For example, someone who scores “Severe” on the QIDS may not score “Severe” on the PHQ-9. This problem could perhaps be solved by using Item Response Theory to identify how scores for frequently used scales measuring each biopsychosocial domain would map onto a common metric (32). This would enable the conversion of scores between psychometric scales, yet would require further validation.

## Using the Automated Clinical Staging Protocol as a Decision Support Tool

Clinical staging is not meant to replace diagnosis but instead act as an adjunct to it. This means that the automated clinical staging protocol presented here, should not (and is not intended to) be taken as the sole indicator of a young person's mental health. Instead, the application of the clinical staging model using HITs provides the opportunity for it to be used as a standardized and scalable decision support tool in services. In tandem with the multidimensional intake assessment administered by the HIT, the automated clinical staging protocol provides a useful heuristic for distilling the complex assessment results into a clinical stage that can be used to help clinicians decide about the best care plan and pathway for a young person.

The ability to assess and process this information prior to the first appointment could expedite the intake process and improve service efficiencies, such as wait times for assessment and treatment, by shifting initial assessment to HITs. Thus, the first application of this tool may be to help differentiate between those who would be suitable for self-directed or low intensity services (stage 1a) from those who require higher intensity services (stage 1b and 2+). Young people at stage 1b or stage 2+ typically require more complex interventions (17) and further assessment (including neuropsychological and circadian), however young people at stage 1a, typically experience milder symptoms and have a lower risk of progressing to a discrete disorder (23). This provides services with an opportunity to direct these young people to suitable care faster by leveraging HITs and the widespread availability of brief online mental health interventions such as Internet-delivered cognitive behavioral therapy (CBT) and other app-based programs (17, 29). This has the dual benefit of ensuring that limited mental health specialist resources directly assist those most at need while also ensuring that those with a relatively lower risk of illness progression receive the appropriate supports for recovery. Importantly, these young people (stage 1a) would not be sent away from the service, but rather HITs are used here to keep the young people in contact with the service so that their progress can be monitored, and if required, they can be directed to higher intensity interventions if they do not respond to the initial treatment options.

As clinical staging operates on a consensus model, a clinical stage suggested by this protocol represents one source of information that the clinician could use when considering a young person's case. For those at higher clinical stages or not accessing self-directed or low intensity services, their cases would ideally be reviewed at a full multidisciplinary team meeting or by a highly skilled clinician to determine the right care plan and pathway (29). To facilitate this staging process, the HIT was designed to collate much of the relevant information in one place and provide an overview of the factors driving the clinical staging decision. With this information, decisions can be made about the need for further assessment, the care team required to address their needs and the overall intensity of services (i.e., type of service and treatments, minimum duration of care required, treatment frequency). This ensures that young people receive the right level of care for an appropriate amount of time to optimize treatment and prevention outcomes (27).

**Figure 2:**
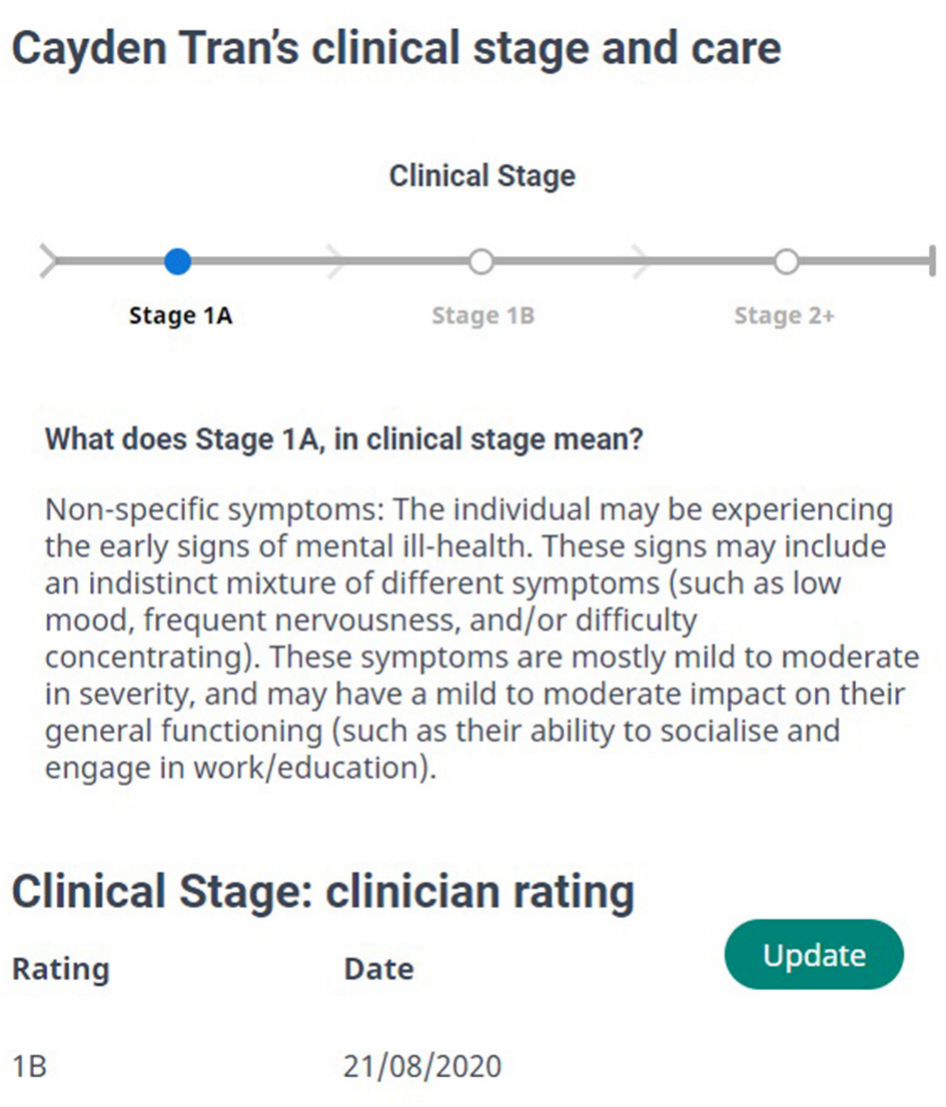
A (fictional) young person's suggested clinical stage from our automated protocol, and the clinician's initial assessment of clinical stage (in this case disagreeing with the protocol).

Ideally, in all cases, after the young person completes the multidimensional assessment a clinician would review their full intake data [including demographic information, assessment responses via a “dashboard” (28), and other contextual information collected during intake] alongside the suggested clinical stage, and record whether they agree with the automated protocols' recommendation (Figure 2). Any cases of disagreement should be referred to a multidisciplinary team for review and care allocation.

In practice, the application of this protocol extends beyond the initial care allocation process and can be applied dynamically over the course of a person's care. A young person's clinical stage should be reviewed periodically over time and HITs can be used to automatically schedule these according to a young person's clinical stage (i.e., stage 1b after 1 month). These reviews integrate further information, including continual multidimensional data from the young person (which would provide clinically valuable information on longitudinal trends) and results from further assessment (e.g., neuropsychological or circadian) if required. These results can then be used to increase confidence in the clinical stage result or identify what pieces of information are required to reduce uncertainty in the result. The dynamic use of this protocol ensures that an individual's care plan is adjusted over time to match any changes in their clinical stage or care needs.

## Discussion and Future Directions

The demand and need for youth mental health care continues to increase, which puts pressure on an under resourced system, and impacts the overall quality of care a young person receives (33). Innovations to the way services assess, treat and monitor youth mental health problems are critical to improve service efficiencies and clinical outcomes.

Young people presenting for youth mental health care typically vary in terms of the type, severity, and complexity of illness. This makes the initial assessment and care allocation process difficult (34), particularly when greater demand forces services to place individuals on waitlists before initial and standard consultations (35). The use of HITs facilitates the initial assessment of a young person's needs prior to face-to-face contact, yet a current limitation of these HITs is being able to distill a comprehensive assessment into useful information for clinical decision making. Current solutions typically heavily rely on scoring of validated measures to identify a young person's symptom severity or need for care; however these approaches do not properly account for the complexity of youth mental health.

In contrast, the automated clinical staging protocol developed and presented here extracts critical clinical information from the multidimensional assessment to provide clinically meaningful evaluation and interpretation of a young person's current mental health illness trajectory. Its proposed use to identify young people in the very early stages of illness (stage 1a) and direct them to the relevant online services for treatment, has the potential to improve current wait times in services for all young people. In addition to improving care pathways, the allocation of care plans according to clinical stage has the potential to guide the type, intensity and duration of treatments according to a young person's illness trajectory.

While, clinical staging has been validated (24–26, 36), the automated clinical staging protocol has yet to be validated within a clinical setting (i.e., does the application of these concepts result in an improved delivery of mental health care and improved mental health outcomes for young people). Hence, an important next step will be to conduct this clinical validation to determine the actual predictive power in the sample of young people attending the mental health services that use the HIT (28). Further work will need to also evaluate the applicability of this automated protocol across different culturally and linguistically diverse populations to determine its generalizability.

As health systems embrace digital health, the medicolegal, and ethical guidelines for the legal and ethical use of health data, algorithms, and clinical decision support tools more generally is critical. It is important that all young people, clinicians, and service managers have a clear understanding of what personal and health information is being collected and how this data will be shared and used. This ensures transparency about how the decision support tool works and generates an output, which will be critical to managing any biases in any algorithm or tool using data to make recommendations about treatment. For this, engaging young people and clinicians in the further design, implementation and roll out of this tool is important for its real-world applicability.

Finally, one of the challenges in the broad implementation of this work (and the wider clinical staging framework to deliver technology-enabled mental health care) is the sector's relative lack of integration with technology, such as HITs. While a consequence of COVID-19 has been the rapid digitization of mental health service provision (12), this needs to continue in order to provide a base from which to execute the described service delivery model in a sustainable and scalable manner. It is important that this continual integration [for example implementing the capability for long term monitoring; (37)] always keeps mental health consumers at the center of their care and promotes their best interests.

Here, we have presented the translation of the clinical staging model into a decision support tool using HITs and its potential use in youth mental health services. The automated clinical staging protocol leverages the clinical validation and evidence base of the staging model to improve its standardization, scalability, and utility by deploying it using HITs. Its use has the potential to enhance clinical decision-making and transform existing care pathways, but further validation and evaluation of the tool in real-world settings is needed.

## Data Availability Statement

The original contributions presented in the study are included in the article/supplementary material, further inquiries can be directed to the corresponding author/s.

## Author Contributions

FI and VC wrote the manuscript. FI, SC, VC, and HY developed the automated staging protocol. TD, ES, and IH supervised the work and provided scientific leadership. All authors assisted with manuscript drafting and approved the final manuscript.

## Conflict of Interest

IH was an inaugural Commissioner on Australia's National Mental Health Commission (2012–2018). He is the Co-Director, Health and Policy at the Brain and Mind Centre (BMC) University of Sydney. The BMC operates an early-intervention youth services at Camperdown under contract to headspace. He is the Chief Scientific Advisor to, and a 5% equity shareholder in, InnoWell Pty Ltd. InnoWell was formed by the University of Sydney (45% equity) and PwC (Australia; 45% equity) to deliver the $30 M Australian Government-funded Project Synergy (2017–2020; a 3-year program for the transformation of mental health services) and to lead transformation of mental health services internationally through the use of innovative technologies. ES is the Medical Director, Young Adult Mental Health Unit, St Vincent's Hospital Darlinghurst, Discipline Leader of Adult Mental Health, School of Medicine, University of Notre Dame, Principal Research Fellow, Brain and Mind Centre, The University of Sydney and Consultant Psychiatrist. She has received honoraria for educational seminars related to the clinical management of depressive disorders supported by Servier and Eli-Lilly pharmaceuticals. She has participated in a national advisory board for the antidepressant compound Pristiq, manufactured by Pfizer. She was the National Coordinator of an antidepressant trial sponsored by Servier. The remaining authors declare that the research was conducted in the absence of any commercial or financial relationships that could be construed as a potential conflict of interest.

## Publisher's Note

All claims expressed in this article are solely those of the authors and do not necessarily represent those of their affiliated organizations, or those of the publisher, the editors and the reviewers. Any product that may be evaluated in this article, or claim that may be made by its manufacturer, is not guaranteed or endorsed by the publisher.
